# Effects of team‐based learning about postpartum haemorrhage on learning outcomes and experience of midwifery students in Indonesia: A pilot study

**DOI:** 10.1002/nop2.623

**Published:** 2020-09-17

**Authors:** Yunefit Ulfa, Yukari Igarashi, Kaori Takahata, Shigeko Horiuchi

**Affiliations:** ^1^ Graduate School of Nursing Science St. Luke’s International University Tokyo Japan; ^2^ Shonan Kamakura University of Medical Sciences Kanagawa Japan

## Abstract

**Aim:**

This pilot study aimed to evaluate the effects of team‐based learning about postpartum haemorrhage on the learning outcomes and experience of midwifery students in Indonesia.

**Design:**

One‐group pre‐test–post‐test study.

**Methods:**

This study enrolled 64 midwifery students as participants from an Indonesian health polytechnic school. This group attended two team‐based learning class sessions (90 min weekly for 2 weeks) on postpartum haemorrhage. Student learning outcomes and experience were assessed quantitatively.

**Results:**

The mean knowledge score (0–100) was significantly higher at post‐test (mean = 85.9, *SD* 9.8) than at pre‐test (mean = 61.4, *SD* 12.9) (*p* < .001). There was a significant difference in the mean clinical reasoning score (12–60) between post‐test (mean = 35.4, *SD* 5.8) and pre‐test (mean = 21.3, *SD* 7.9) (*p* < .001). Most students (98.4%) engaged in classroom activities.

## 1 INTRODUCTION

Midwifery education is a reflection of the educators’ academic background in terms of the adequate development and implementation of a curriculum or the self‐development of knowledge and skills management (Botma & Nyoni, 2015). Midwifery educators in Indonesia provide academic instruction using a traditional didactic lecture approach. This approach was cited as the cause of the low quality of knowledge and clinical reasoning of midwifery students, leading to inflexibility in midwifery practice (Shields & Hartati, 2003; World Bank, 2015; Yanti, Claramita, Emilia, & Hakimi, 2015). However, large classes, few instructors and time limitations have understandably tied the educators to the traditional learning approach. More importantly, the educators in most Indonesian nursing institutions may not be prepared educationally and experientially to implement more innovative teaching approaches (Edwards, 2012).

## 2 BACKGROUND

The transition from traditional lecture, which is centred on the instructor, to active learning to engage students has become a main concern in professionalizing maternity care. Previous studies have reported that to be able to manage problems in maternity care, nurses and midwives need to have problem‐solving and critical thinking skills to develop their clinical reasoning (Currey, Oldland, Considine, Glanville, & Story, 2015; Kim, Song, Lindquist, & Kang, 2016). The traditional learning method, which conditions students to passively receive information, does not support the above requirements as it does not actively stimulate and explore the students’ potentials in learning (Lee, 2018). By comparison, the teaching methods of active learning enhance the critical thinking and clinical reasoning of students. Positive outcomes of active learning methods such as cognitive attainment and attitudinal and behaviour outcomes have been identified in numerous studies of nursing and midwifery education (Abdullah, Ullah, & Bano, 2017; Dearnley, Rhodes, Roberts, Williams, & Prenton, 2018; Lee, 2018).

Midwives as professional healthcare providers desire the integration of more knowledge and critical thinking to enhance clinical reasoning. Therefore, the application of course concepts to real complex scenarios is needed based on a learning method that encourages students to deepen learning and develop their critical thinking skills. Furthermore, interprofessional skills such as communication, teamwork and self‐leadership are required for leading a health service in the community. A pedagogical method that appears to be appropriate in promoting these skills is team‐based learning (TBL), as it points out the strength of teamwork and communication along with the integration of information to assess the concept application. Additionally, it is compatible with the typical midwifery schools in Indonesia, which still have small numbers of faculty (Ministry of Research, Technology, & Higher Education, Indonesia, 2019).

In TBL, one facilitator gives a line of strategic instructions to students for them to perform several individual and team activities, which motivate them to participate actively in learning (Michaelsen, Parmelee, McMahon, & Lavine, 2008). The structured activities inside and outside the class spur students to maximize their learning potential, teamwork and communication skills (Kim et al., 2016). Therefore, TBL is considered to promote the growth of professionalism and confidence (Cheng, Liou, Hsu, et al., 2014; Considine, Currey, Payne, & Williamson, 2014; Currey et al., 2015).

In the clinical setting, midwives must manage maternal–neonatal emergencies, particularly postpartum haemorrhage (PPH). PPH is an important and serious condition and accounts for 27% of maternal mortality in countries across the globe (Say et al., 2014) and 20% in Indonesia (National Institute of Health Research & Development, Ministry of Health, Republic of Indonesia & United Nations Population Fund, 2012). The Indonesian government has the difficult task of decreasing this PPH problem by increasing the number and quality of midwives attending to childbirth and who are skilled in managing PPH (Ministry of Health, Indonesia, 2015; Shankar et al., 2008).

To the best of our knowledge, there has been no study on the application of TBL to midwifery students in Indonesia. In particular, a TBL programme about PPH has not yet been apparently conducted to date. This pilot study aimed to assess the effects of TBL about PPH on midwifery students’ (a) learning outcomes as measured in terms of knowledge and clinical reasoning (primary outcomes) and (b) learning experience as measured using classroom engagement survey (CES) and team‐based learning Student Assessment Instrument (TBL‐SAI).

### 2.1 Research questions

1. What are the effects of TBL about PPH on the learning outcomes (i.e. knowledge and clinical reasoning) of midwifery students in a health polytechnic school in Indonesia?
2. What are the effects of TBL about PPH on the learning experience of midwifery students at a health polytechnic school in Indonesia?

#### 2.1.1 Operational definition

Active learning involves learning activities that stimulate students to become more engaged in their study, which leads to a broader perspective about what they are doing (Hyun, Ediger, & Lee, 2017). For the purposes of this study, active learning strategies were defined as any instructional methods that engage students actively in the learning process.

## 3 METHODS

### 3.1 Design

The study used a one‐group pre‐test–post‐test design.

### 3.2 Outcomes

The primary outcomes were learning outcomes (i.e. knowledge and clinical reasoning). Knowledge was measured at three time points (i.e. pre‐test, post‐test and 2 weeks post‐test), and clinical reasoning was measured at two time points (i.e. pre‐test and post‐test).

The secondary outcome was learning experience (as measured using CES and TBL‐SAI). CES was carried out twice after the TBL class session and TBL‐SAI was used one time after the second TBL class session was finished. The potential confounders were age and the students’ previous education, which were included in the demographic data of the questionnaire.

### 3.3 Setting

This pilot study was conducted in a department of midwifery at an Indonesian health polytechnic school in West Sumatera, Indonesia. This school is the oldest midwifery educational institution in Central Sumatera. Data were collected between January 2019 and March 2019.

### 3.4 Participants

The sample size was determined by considering the primary outcomes using G*Power 3.1, with an effect size of 0.4 at a power of 80%; the alpha level was 0.05 with two dependent means in the *t* test; the estimated sample size was 41. By considering a dropout rate of 20%, the total sample size was estimated to be 50. Thus, the minimum sample size of students is 50 (Faul, Erdfelder, Buchner, & Lang, 2009).

Second‐year diploma level midwifery students were invited to participate in the study. The inclusion criteria were as follows: (a) have no experience of TBL; (b) graduated from a senior high school (without a nursing background); and (c) completion of the previous semester. The exclusion criteria were as follows: (a) have prior experience of TBL; (b) graduated from a nursing school; and (c) non‐completion of the previous semester.

### 3.5 Instruments

The data collection tools used were the pre‐test and post‐test knowledge and clinical reasoning of PPH, 2 weeks post‐test (knowledge retention), CES after each TBL session is completed and TBL‐SAI after the completion of the TBL sessions. Students who consented to participate in this study were given their own identification number, which was written in the answer sheet instead of their name.

### 3.6 Knowledge of PPH

The researchers of this study developed a 15‐item multiple‐choice questionnaire based on the national midwife competency test in Indonesia and the textbook and National Handbook for Managing PPH in Indonesia (Ministry of Health, Indonesia, 2014). Two experts on this topic verified the content validity of the tool. The total score ranged from 0–75 and was mathematically transformed to 0–100 to be in alignment with the grading system of the school. Higher scores indicate good knowledge attainment.

### 3.7 Clinical reasoning

The researcher (YU) used four items (i.e. 1, 4, 5 and 6) of the 10‐item Clinical Reasoning Evaluation Simulation Tool developed by Liaw et al. (2018) and adjusted for the present study. The vignette was selected as the relevant tool for healthcare professionals to develop clinical reasoning (Carvalho, Oliveira‐Kumakura, & Morais, 2017) and applied to the present study. The researcher developed three vignettes of PPH. The five‐point scale developed by Liaw et al. (2018) with the description of each score was used. The potential scores ranged from 12–60. Higher scores indicate better clinical reasoning and vice versa. Two midwifery educators from the school marked the students’ answers using the scoring as noted. The educators worked in separate rooms and checked each student's answers. The results of both educators were calculated, and the mean scores were obtained.

### 3.8 Classroom engagement survey

CES contains eight items for measuring learner participation during the class (Baylor College of Medicine, 2001). The questionnaire instructs the participants to rate the session that has just been completed. The items are scored on a five‐point Likert scale; the maximum score ranged from 5–40; higher scores indicate greater engagement. The CES Cronbach's alpha was 0.881 established with undergraduate nursing students (Mennenga, 2013).

### 3.9 Team‐based learning student assessment instrument

Mennenga (2013) developed the TBL‐SAI. For the 33‐item SAI, the five‐point Likert scale is scored from 1 (*strongly disagree*)–5 (*strongly agree*). TBL‐SAI has three subscales: Accountability, Preference and Satisfaction. As for the Accountability subscale, it indicated whether the students perceived themselves to be more prepared before attending the class. The Preference subscale showed whether the students favoured a TBL class and want to have more classes using the TBL method. For the Satisfaction subscale, it reported whether the students had positive results and enjoyed the TBL session. Branney and Priego‐Hernández (2018) established its internal consistency (α = 0.88) with second‐year undergraduate nursing students from a UK institution.

### 3.10 Data collection and procedure

After obtaining permission to collect data in December 2018, the researcher and research assistant verbally provided details of the study, as well as the inclusion and exclusion criteria to second‐year midwifery students and also posted the information on the school's communication board in January 2019. Those agreeing to participate in the study signed the informed consent after being informed that non‐participation in the research would not influence their academic grade. The students who declined to participate signed the refusal form.

PPH is one of the 10 topics taught in maternal–neonatal emergency. The other nine topics are taught by traditional lectures and seminars. Initially, the topic content was designed by the researcher in accordance with the topic objectives. Based on the learning objectives, the application exercise and team Readiness Assurance Test (tRAT), which also served as the individual RAT (iRAT), were developed. Pre‐reading articles that supported the necessary knowledge were selected. The pre‐reading materials, RAT and application exercises were approved by two faculty content experts.

The intervention was performed three times, one for preparation and two for the TBL class from January 2019 to February 2019. A week prior to the actual TBL class, students attended a TBL preparation day to do the following: (a) discuss the TBL process; (b) divide the group into teams of 5–6 students; (c) schedule appointments; (d) provide PPH pre‐reading materials and references; (e) instruct students to prepare before attending the class next week; and (f) do a pre‐test. The TBL class was conducted twice, once a week for 90 min. In the last day of the TBL class, the students completed the post‐test. Two weeks after the TBL class, the students took the follow‐up test. The data collection was completed in March 2019.

At the TBL session, the researcher acted as a facilitator and explained the aim and overview of the lesson. Thereafter, the students took the iRAT without using notes, books and other resources. The iRAT was composed of 10 multiple‐choice questions. After completing and submitting their answer sheets, the students then took the tRAT. During the tRAT, the students discussed the answers within their small groups. They chose answers using an Immediate Feedback Assessment Technique. Following the tRAT, the group could choose to submit a written appeal (if needed) for their answers to a question with supporting references or seek clarification for unclear questions and the facilitator would provide clarification. If the appeal were accepted, then the group would get an additional grade. After the tRAT session, the instructor gave a minilecture based on the five low‐scoring questions. Finally, an application exercise using a vignette of concepts discussed with the group. The students summarized their case and reported their answers to the whole class. This was followed by inter‐team debates and discussion. The process remained the same for the next class (Table 1).

**TABLE 1:** Team‐based leaning programme of the postpartum haemorrhage protocol consisting of a two‐session educational programme

Content/time		Subtopic
Preparation (30 min)	Discuss the TBL process (20 min) Form groups (5 min) Provide learning materials and references (5 min)	
TBL session 1 (90 min)	TBL Overview (5 min) iRAT (10 min) tRAT (10 min) Appeal (10 min) Minilecture (15 min) Application exercise (35 min) Feedback (5 min)	Haematologic changes in pregnancy Uterine atony Retained placenta
TBL session 2 (90 min)	Overview (5 min) iRAT (10 min) tRAT (10 min) Appeal (10 min) Minilecture (15 min) Application exercise (35 min) Feedback (5 min)	Perineal injury Retained part of placenta Uterine inversion Endometritis Shock hypovolaemic Emergency communication

Abbreviation: min, minutes.

### 3.11 Data analysis

Demographic data, students' knowledge of PPH, clinical reasoning, CES results and TBL‐SAI results were analysed using descriptive statistics in terms of percentages, means and standard deviations. The chi‐square goodness‐of‐fit test was used to determine the sample distribution of the demographic data. The normality of the data was assessed using the Kolmogorov–Smirnov test. The differences in the mean knowledge scores between pre‐test and post‐test, pre‐test and 2 weeks post‐test and post‐test and 2 weeks post‐test were analysed using the paired *t* test. The differences in mean clinical reasoning scores and CES were analysed using the paired *t* test. *p*‐values <0.05 were considered to indicate a statistically significant difference. Statistical analysis was performed using the Statistical Package for the Social Sciences (SPSS) 22.0 for windows. The item analysis by Hopkins and Antes (1990) was used for the analysis of iRAT.

### 3.12 Ethical considerations

This study was conducted based on the ethical guidelines of harmlessness, voluntarily participation and protection of privacy and personal information. Ethical approval was obtained from the IRB of the Ethics Committee of St. Luke's International University, Japan (Number 18‐A062).

## 4 RESULTS

### 4.1 Demographic characteristics

There were 69 eligible students for this study. Of these 69 students, four students declined to participate, resulting in 65 students consenting to participate. Of these 65 students, one student was excluded because of insufficient data. Finally, the study was started with 64 students as participants (98.5%). The data and baseline demographics of these 64 students enrolled were analysed. Table 2 shows the demographics of the participants with no significant differences in the demographic variables among the groups.

**TABLE 2:** Demographics of participants (*N* = 64)

	*N* (%)	*x* ^2^	*p*‐value
Age (years)			
19	32 (50.0)	1.32	.52
20	23 (35.9)		
>20	9 (14.1)		
Education			
Islamic Senior High School (Specific)	8 (12.5)	2.25	.13
Senior High School (General)	56 (87.5)		
Future plan to be a clinical midwife			
No	20 (31.3)	0.05	.83
Yes	44 (68.8)		
Previous Grade Point Average (GPA)			
Cum laude (3.51–4.00)	29 (45.3)	0.56	.45
Very satisfactory (2.76–3.50)	35 (54.7)		

### 4.2 Primary outcomes: knowledge and clinical reasoning with regard to postpartum haemorrhage

**FIGURE 1:**
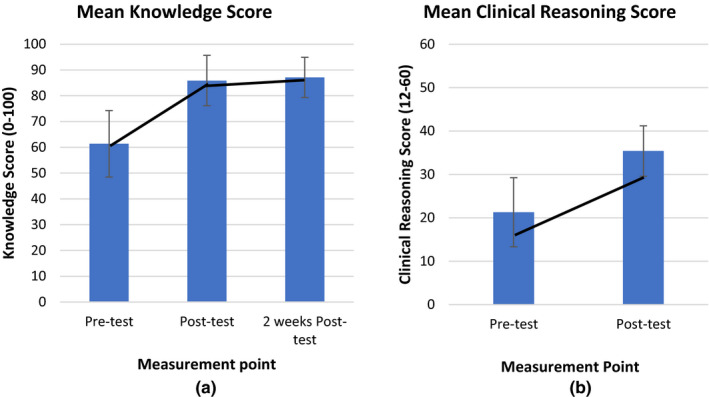
(a) Comparison of mean knowledge scores and (b) mean clinical reasoning scores

The pre‐test mean knowledge score was 61.4 (*SD* 12.9); the post‐test mean knowledge score was 85.9 (*SD* 9.8); the 2 weeks post‐test mean knowledge score was 87.1 (*SD* 7.8). (Figure 1) The *t* test revealed a significant difference in the mean knowledge score between pre‐test and post‐test (*t* = −12.4, *p *<* *.001) and between pre‐test and 2 weeks post‐test (*t* = −15.68, *p* < .001). For the clinical reasoning, the pre‐test and post‐test mean scores were 21.3 (*SD* 7.97) and 35.4 (*SD* 5.81), respectively, showing a significant increase in the clinical reasoning score (*t* = −13.8, *p* < .001) (Figure 1).

### 4.3 Secondary outcome: student learning experience (classroom engagement survey and team‐based learning student assessment instrument)

Almost all the CES questions had a good response (> 3). However, there was a slight decrease in the CES mean score from CES 1–CES 2. Taken together, the overall results indicate that most students were engaged with the class (Table 3).

**TABLE 3:** Comparison of the mean scores of the classroom engagement survey items

	Item question	TBL class 1	TBL class 2	*t*	*p*‐value
		Mean (*SD*)	Mean (*SD*)		
Q1	Most students were actively involved	4.36 (0.7)	4.33 (0.6)	0.31	.76
Q2	I had fun in class today	4.48 (0.6)	4.44 (0.6)	0.49	.63
Q3	I contributed meaningfully to class discussions	4.02 (0.7)	3.92 (0.7)	0.92	.36
Q4	Most students were not paying attention ®	4.14 (0.6)	4.02 (0.7)	1.43	.16
Q5	I paid attention most of the time	4.34 (0.7)	4.25 (0.7)	1.06	.29
Q6	I did not enjoy class today ®	4.53 (0.5)	4.33 (0.6)	2.42	.02
Q7	I participated in the class most of the time.	3.81 (0.7)	3.77 (0.7)	0.45	.65
Q8	I would like more class sessions to be like this one.	4.52 (0.6)	4.30 (0.6)	2.58	.01
	Total	34.2 (*SD* = 3.4)	33.3 (*SD* = 3.7)	1.78	.08

Note.

For the CES score, 24 is neutral (Baylor College of Medicine, 2001). A higher score indicates that the students were more engaged in the classroom.

For the next measurement of learning experience, TBL‐SAI (“Accountability,” “Preference” and “Satisfaction”) was used to assess the student's experience of TBL. The “Accountability” mean score was 32.3 (*SD* 3.7), and all of the students (100%) had the same mean scores or above neutral (24.0). This result indicated that the students were more prepared and had a high contribution to their teams.

The “Preference” mean score was 56.7 (*SD* 5.9), and 98.4% of the students had the same mean scores or above neutral (48.0). Only one student (1.6%) had a mean score below neutral. These results indicated that most students favoured TBL.

The “Satisfaction” mean score was 39.0 (*SD* 3.9). All of the students (*N* = 64) had the same mean scores or above neutral (27.0). These results indicated that the students enjoyed the TBL session.

Overall, the TBL‐SAI mean score was 128 (*SD* 10.6), with the mean scores of all the students the same or above neutral (99.0). This result suggested that the students had a positive experience with TBL.

### 4.4 Practicality of team‐based learning programme

This programme was accepted by the students, and there were no students who dropped out after starting the intervention. According to the results of CES 1 and CES 2, most students had a positive attitude towards TBL. Further support for the acceptability of TBL comes from the positive responses in the TBL‐SAI questionnaire. Some examples include, “I enjoy team‐based learning activities” and “Team‐based learning activities are fun” which had a higher score (> 4.00). Notably, the programme was also on demand as indicated in a CES response, “I would like more class sessions to be like this one” (*mean *=* *4.41; *SD* 0.64) and students had a high preference for TBL (98.4%) as shown by the TBL‐SAI.

There were no adverse events during the intervention. The 90‐min session time was maintained. The item analysis (Hopkins & Antes, 1990) of RAT 1 and RAT 2 indicated items that needed to be revised (Table 4) because the difficulty level was too low or high and revisions in the discrimination level were needed. Regardless, the researcher must carefully revise or substitute questions because they tap into the learning objectives which students must master.

**TABLE 4:** Item analysis of iRAT 1 and iRAT 2

Questions	Difficulty	Difficulty level	Discrimination	Discrimination level	Conclusion
iRAT 1					
Q1. Blood volume percentage	0.78	Medium	0.41	Very good items	Good item
Q2. Blood volume in pregnancy	0.73	Medium	0.47	Very good items	Good item
Q3. Definition of postpartum haemorrhage	0.81	Low	0.12	To be discarded	Revised item
Q4. Hypervolaemia of pregnancy	0.32	Medium	0.53	Very good items	Good item
Q5. Causes of secondary postpartum haemorrhage	0.64	Medium	0.59	Very good items	Good item
Q6. Definition of uterine atony	0.95	Low	0.18	To be revised	Revised item
Q7. Signs of retained placenta	0.57	Medium	0.53	Very good items	Good item
Q8. Treatment of retained part of placenta	0.21	High	0.29	To be revised	Revised item
Q9. Treatment of uterine atony	0.2	High	0.12	To be discarded	Revised material
Q10. Placental attachment	0.42	Medium	0.47	Very good items	Good item
iRAT 2					
Q1. Signs of uterine inversion	0.85	Low	0.29	To be revised	Revised item
Q2. Risk factor of endometritis	0.48	Medium	0.47	Very good items	Good item
Q3. Diagnosis of retained part of placenta	0.85	Low	0.35	Very good items	Revised item
Q4. Predisposing factors of perineal injury	0.89	Low	0.29	To be revised	Revised material
Q5. Degree of perineal injury	0.9	Low	0.23	To be revised	Revised item
Q6. Classification of hypovolaemic	0.25	High	0.35	Very good items	Revised material
Q7. Cause of postpartum haemorrhage	0.76	Medium	0.52	Very good items	Good item
Q8. Shock index	0.56	Medium	0.41	Very good items	Good item
Q9. Communication in emergency situation	0.65	Medium	0.65	Very good items	Good item
Q10. Treatment of hypovolaemic shock	0.51	Medium	0.18	To be revised	Revised material

In addition, the application exercise in TBL session 2 needs to be reviewed and revised. Based on the observation made during the class, the application exercise failed to stimulate debate or discussion among the students owing to the simple scenario (vignettes) and less distractors. However, in a TBL‐SAI statement, “I have a positive attitude towards TBL activities,” the mean score was 4.35 (*SD* 0.54). Thus, the intervention was successfully delivered to the students. Moreover, some questions on knowledge also need to be revised based on the item analysis. For the clinical reasoning, there is a need to add a case to facilitate a clear understanding of PPH.

## 5 DISCUSSION

### 5.1 Primary outcomes: knowledge and clinical reasoning of postpartum haemorrhage after the TBL programme

The results showed that there was a significant increase in PPH‐related knowledge between pre‐test and post‐test and a slight increase between post‐test and 2 weeks post‐test. Therefore, the intervention had a positive effect on student knowledge acquisition. These results are consistent with those of previous studies of TBL for nursing students (Branson, Boss, & Fowler, 2015; Harmon & Hills, 2015; Kim et al., 2016; Siah, Lim, Lim, Lau, & Tam, 2019) and medical students (Harakuni, Nagamoti, & Mallapur, 2015; Mody, Kiley, Gawron, Garcia, & Hammond, 2013; Rezaee, Moadeb, & Shokpour, 2016).

The sequence of activities of the TBL process (iRAT, tRAT, appeal process and application exercise) helped students to gain knowledge. The RAT encouraged the students to prepare themselves by reading the material before attending the class, and they used their prior knowledge to open the discussion within their team (Gopalan, Fox, & Gaebelein, 2013). In addition, the appeal activity requires accuracy from the students about the questions posed in the RAT. The students had to discern whether the questions were appropriate or not, and then they proceeded to the application exercise. This process helped the students develop their critical thinking which enables them to engage in clinical reasoning (Okubo et al., 2012).

The mean knowledge score between post‐test and 2 weeks post‐test showed a slight increase, which indicated that the TBL class supported the knowledge retention of the students. This result is supported by research on the learning pyramid (Dale, 1969), showing that discussions have a 50% retention rate compared with lecture (5%) or reading (10%). As the post‐tests were carried out within 2 weeks after the last post‐test, it may be too early to make a conclusion about the sustained knowledge retention; thus, further research is needed to assess long‐term knowledge retention at 1 month and 3 months after the TBL class. Rezaee et al. (2016) conducted a quasi‐experiment study with undergraduate medical students. They reported that students participating in a TBL class earned higher knowledge scores at 2 months post‐test than students participating in a lecture class, although there was no significant difference in knowledge retention between the groups. In addition, Alimoglu, Yardim, and Uysal (2017) and Cevik, Elzubeir, Abu‐Zidan, and Shaban (2019) showed that medical students who were in the TBL group had a higher mean knowledge retention score (after one year) than that of the lecture group.

In contrast, the quasi‐experiment studies conducted by Emke, Butler, and Larsen (2015) and Farland, Barlow, Levi Lancaster, and Franks (2015) demonstrated no significant difference in the knowledge retention scores between students in TBL classes and students in non‐TBL classes after several months. As the interactive learning method, TBL is designed to encourage students to develop deep thinking about the material than a traditional lecture, and further research may be necessary to identify if some contents are better than other contents in promoting knowledge retention using the TBL approach to learning.

For the secondary outcome, the mean clinical reasoning score significantly increased between pre‐test and post‐test. This result is supported by a previous study by Brewer, Hammond, and Ulrich (2013) who showed that TBL strategies improved the clinical reasoning of nursing students. In a related study, Okubo et al. (2012) showed that TBL is useful in improving the clinical reasoning ability of fourth‐year medical students with limited clinical exposure. Two other studies of undergraduate medical students demonstrated that the medical students in the TBL class have a better performance in clinical reasoning (Jost, Brüstle, Giesler, Rijntjes, & Brich, 2017; Tan, Tan, & Ng, 2016).

As regards critical thinking, the case complexity for the application exercise in TBL activities has been shown to present common clinical problems. It fostered students to think critically. The integration of critical thinking in the assessment of a students’ condition should lead to improved clinical reasoning. Instead of memorizing factual knowledge, students were reportedly motivated to think critically and solve problems (Ihm, Shin, & Seo, 2020).

In the present study, the second‐year midwifery students still had limited clinical experience. Using a vignette format is expected to assist the students in responding more appropriately when they practice at a maternity clinic or hospital. The clue in the scenario led the midwifery students to discuss further with their team. This enables them to project the events that they might encounter during their clinical practice. The application exercise is anticipated to help the students obtain accurate information, observe signs and symptoms, interpret and make a diagnosis before identifying the most appropriate intervention that will improve the students’ condition.

Managing obstetric emergencies is critical for midwives. The knowledge and clinical reasoning questions covering the diagnosis, causes, risk factors and treatment of PPH require complex clinical reasoning. Students must use critical thinking to integrate their knowledge and be able to provide answers on the answer sheet. The evaluation depends on the thorough analysis of information about the case. Regarding PPH, a systematic review covering 29 countries found a low level of PPH identification and management (Finlayson, Downe, Vogel, & Oladapo, 2019). Thus, the application of TBL is expected to increase the ability of midwives to assess and manage PPH. For further research, we need to use a control group to appropriately assess the differences of learning outcomes between a TBL class and a lecture class.

### 5.2 Secondary outcomes: student learning experience of team‐based learning

The student learning experiences were measured using CES and TBL‐SAI. This study found that the midwifery students had a higher CES score. In the TBL activities, students encouraged to speak and act in the learning process since they required to discuss and convey ideas or knowledge they have. For example, in tRAT students need to discuss to get one correct answer, likewise in the appeal process and application exercise. These activities explained the reason why students more engaging in the class. Similarly, Cheng, Liou, Tsai, and Chang (2014) observed that most nursing students perceived TBL to be more engaging than conventional teaching.

Learning experience was also measured using TBL‐SAI. The students reported positive experiences with TBL. The “Accountability” subscale results indicated that the students perceived themselves to be more prepared before the class. Students were motivated to read the materials before attending the class because they had to take the iRAT.

The “Preferences” subscale results indicated that the students favoured the TBL class even though the lecture class also helped them in their study. By contrast, Della Ratta (2015) revealed that undergraduate nursing students found it difficult to adapt to active learning because they were familiar with passive learning. Therefore, some preparations about active learning may be necessary.

The “Satisfaction” subscale results showed that the students had positive results and enjoyed the TBL session. Branney and Priego‐Hernández (2018) used mixed methods with undergraduate nursing students in their applied pathophysiology class and found that the students were more satisfied with the TBL class and that most of the students wanted to make a valuable contribution.

### 5.3 Practicality of TBL programme for PPH

The present findings indicate the successful implementation of TBL among the midwifery students. The acceptability of the TBL programme was demonstrated by the students’ engagement in the class and their enjoyment of the TBL activities. The students expressed their willingness to participate in this programme and their desire to have more TBL classes given the choice. In addition, TBL promoted active learning among the students and made the procedures easy to understand and follow within the allotted timeframe. The positive TBL learning experiences in the resource‐limited teaching settings were consistent with the findings from a previous study of medical education in Zimbabwe (Gray et al., 2014).

Preparing the RAT and application exercise requires accuracy so that both can cover all the learning objectives. Likewise, the RAT questions need to be psychometrically reviewed. The question must display an adequate level of difficulty and discrimination and can be answered within the appropriate time. As Morris (2016) stated, students become tired if it takes a long time to complete the RAT. Moreover, the application exercise must capture actual problems facing health providers in clinical practice. Scenarios should stimulate students’ critical thinking; if the vignette is too simple and has few distractors, it would not encourage deep discussion in the class (Morris, 2016). Therefore, it is important to prepare the application exercise carefully and adjust the time needed to implement the exercise in the TBL class.

Finally, reading resources must be available in the library or accessible to students, particularly if the main resources are not available in the library. To improve access to valuable content, it is important to inform students that other books may have the necessary information. Moreover, the Internet may provide easy access to resources as reading materials (Gray et al., 2014). In addition, for main books or journals that are difficult to obtain, the instructor could write a summary of important concepts and points for the students.

### 5.4 Strengths and limitations

The strengths of this pilot study are its high practicality and potential for conducting a larger study. The TBL programme and its length were appropriate, which can serve as a useful guide for the subsequent study. Additionally, the primary and secondary outcomes are well designed and can be used for the subsequent study.

Regarding the study limitations, we could not definitely ascertain whether the follow‐up test was conducted only 2 weeks after the intervention. As for the next study, the follow‐up test was conducted 1 month and 3 months after the last course. Furthermore, there is a need to revise some of the items in the iRAT as well as the knowledge question as they do not meet the item analysis criteria. There is also a need to add a case for the clinical reasoning test to achieve a clear understanding by the students. Finally, the materials for the main research can still be improved.

## 6 CONCLUSION

The present pilot study found that TBL about PPH improved the learning outcomes (i.e. knowledge and clinical reasoning) of midwifery students in a health polytechnic school in Indonesia. Moreover, the TBL class increased the engagement and satisfaction of the midwifery students in the classroom, indicating a positive learning experience. Additionally, the developed TBL protocol facilitated understanding of PPH among the midwifery students and may therefore be a promising programme for developing a subsequent larger experimental study.

## CONFLICT OF INTEREST

The authors declare that they have no conflicts of interest associated with this study.

## AUTHORS’ CONTRIBUTIONS

YU, YI, KT and SH: Study design. YU: Data collection. YU, KT and SH: Data analysis. YI, KT and SH: Study supervision. YU, YI, KT and SH: Manuscript writing. KT and SH: Critical revisions for important intellectual content.

All authors have read and approved the final manuscript.

## Data Availability

All raw data generated during and/or analysed during this study are available from the corresponding author on reasonable request.
